# A systematic review on the possible role of microRNA-155 in cancer treatment

**DOI:** 10.3389/fphar.2026.1885427

**Published:** 2026-10-20

**Authors:** Shruthi Nagainallur Ravichandran, Antara Banerjee, Pier Paolo Parnigotto, Surajit Pathak, Silvia Barbon

**Affiliations:** 1 Medical Biotechnology Lab, Faculty of Allied and Healthcare Sciences, Chettinad Academy of Research and Education, Chettinad Hospital and Research Institute, Chennai, Tamil Nadu, India; 2 Foundation for Biology and Regenerative Medicine, Tissue Engineering and Signaling – T.E.S. (ETS), Padua, Italy; 3 Department of Neuroscience, Section of Human Anatomy, University of Padua, Padua, Italy

**Keywords:** cancer, chemotherapy, drug resistance, drug sensitivity, microRNA-155 (miR-155), radiotherapy, targeted therapy

## Abstract

**Background:**

Overexpression of microRNA-155 (miR-155) has been associated with cancer development, metastasis, and possible drug resistance. This systematic review analyzed the evidence from the existing literature on miR-155 expression in cancer patients undergoing treatment.

**Methods:**

A thorough literature search was conducted in databases and search engines (ProQuest, PubMed, and Google Scholar) from January 2019 to December 2024 to identify clinical evidence on miR-155 expression in cancer treatments. To assess miR-155 expression in relation to clinical outcomes, we included 15 cancers in this systematic review. Following the PRISMA 2020 guidelines, three independent reviewers selected, extracted, and assessed the data against the predefined inclusion criteria, and a fourth reviewer verified the assessments and resolved discrepancies.

**Results:**

Of the 15 cancers analyzed, 19 eligible studies covering eight cancers were identified and included in the review, namely breast cancer, non-small cell lung cancer, pediatric acute lymphoblastic leukemia, cutaneous melanoma, non-muscle-invasive bladder cancer, papillary thyroid cancer, pancreatic ductal adenocarcinoma, and diffuse large B-cell lymphoma. Screening and evaluation of these 19 studies showed that miR-155 expression varied by cancer type, treatment strategy, and patient sample type. While most studies associated miR-155 overexpression with treatment resistance, a few reported an association between elevated miR-155 expression and enhanced treatment sensitivity. A limitation of this study is that the search for studies reporting miR-155 expression in relation to therapeutic outcomes in patients with cancer was restricted to three databases and a 5-year timeframe.

**Conclusion:**

Overall, the results of this study indicate that the prognostic and predictive significance of miR-155 warrants further evaluation.

**Systematic Review Registration:**

PROSPERO ID, CRD42025632573.

## 1 Introduction

Approximately 20 million new cases of cancer are diagnosed worldwide each year, and this number is rising, possibly due to aging and lifestyle changes (Bray et al., 2024). Recent global cancer statistics indicate that lung, breast, colorectal, pancreatic, prostate, stomach (gastric), liver, thyroid, cervical, bladder, non-Hodgkin lymphoma, oesophageal, and leukemia contribute significantly to the global cancer burden (Di Martino et al., 2022; Sedeta et al., 2023; Bray et al., 2024). Other frequently diagnosed cancers that contribute considerably to this burden include oral cancer and melanoma (skin cancer), whose incidence varies geographically due to differences in risk factors and access to screening (Bray et al., 2024; Coletta et al., 2024; Wang et al., 2025). Together, the high mortality associated with these aggressive cancers and the increasing challenge of drug resistance highlight the need for continued research to improve global cancer prevention and management.

Current cancer treatment strategies include surgery, chemotherapy, radiotherapy, targeted therapy, immunotherapy, and combined treatments tailored to the tumor type and the molecular profile of each patient’s cancer (Zafar et al., 2025). Chemotherapeutics such as platinum-based compounds, antimetabolites, and taxanes remain widely used, alongside targeted drugs and immune checkpoint inhibitors (Sazonova et al., 2021; Yusoh et al., 2025). Despite improved outcomes, treatment resistance remains a major clinical challenge (Bota et al., 2024; Yusoh et al., 2025). This is mostly due to genetic alterations, dysregulated signaling pathways, enhanced DNA repair, altered drug metabolism, and epigenetic or microRNA (miRNA)-mediated mechanisms (Lei et al., 2023).

Recent findings suggest that miRNAs play a major role in the molecular mechanisms underlying multidrug resistance (Pavlíková et al., 2022). Additionally, aberrant miRNA expression has been correlated with resistance to chemotherapy and radiotherapy (Kousar et al., 2022; Al-Hawary et al., 2024). According to the existing literature, several miRNAs including miR-10b, miR-196a, miR-371, miR-372, miR-373, miR-302, miR-17, miR-18a, miR-19a, miR-19b, miR-20a, miR-92a, miR-15a, miR-16-1, miR-125b, miR-9, miR-21, miR-34a, miR-200c, miR-486-3p, miR-4443, miR-4488, miR-204-5p, miR-199b-5p, miR-222, miR-340, the let-7 family, miR-4458, miR-146a, miR-205-5p, miR-216a, miR-150, miR-483-5p, miR-454-3p, miR-31-5p, miR-613, miR-181a, the miR-449 family (miR-449a/b/c), miR-148a-3p, miR-100-5p, miR-99-5p, and microRNA-155 (miR-155) may play a role in chemotherapy and radiotherapy resistance (Wang Z. et al., 2024; Attathikhun et al., 2025).

miR-155, encoded by the miR-155 host gene (miR-155HG), has been shown to regulate several biological processes, including immune responses, inflammation, cancer progression, and drug resistance (Pathak et al., 2015a; Kalkusova et al., 2022; Qian et al., 2024). miR-155 refers to the miRNA precursor family, whereas miR-155-5p and miR-155-3p denote the mature miRNAs originating from the 5ʹ and 3ʹ arms of the precursor hairpin. Notably, miR-155 functions predominantly as an oncogenic miRNA (oncomiR) that targets tumor suppressor genes and promotes cancer by modulating proliferation, migration, invasion, epithelial-mesenchymal transition, angiogenesis, stemness, and apoptosis (Pathak et al., 2015b; Raue et al., 2021; Aghiorghiesei et al., 2025). It has also been reported that overexpression of miR-155 is associated with metastasis and drug resistance in gastric and breast cancers (Qian et al., 2024). However, recent studies highlight a dual role of miR-155, suggesting that its overexpression may enhance sensitivity to certain anticancer therapies and inhibit tumor growth under specific therapeutic conditions (Wang et al., 2022; Qian et al., 2024; Shen et al., 2024; Hussain et al., 2025).

This systematic review therefore aimed to assess miR-155 expression across 15 cancers based on previously published clinical studies and to evaluate its association with treatment outcomes, including treatment resistance, treatment sensitivity or response, and survival outcomes following chemotherapy, radiotherapy, and other therapeutic modalities.

## 2 Methodology

### 2.1 Study protocol

This systematic review was registered in January 2025 in the International Prospective Register of Systematic Reviews (PROSPERO) ID: CRD42025632573.

### 2.2 Information sources

To identify relevant articles, a comprehensive literature search was conducted in databases and search engines, including Google Scholar, ProQuest, and PubMed, from 01-January-2019 to 31-December-2024. Given the significant advances in miRNA profiling and its use in cancer treatment, this systematic review aimed to analyze the most recent clinical evidence. Limiting the search for studies on miR-155 expression in cancer to a 5-year timeframe may have reduced methodological variation across studies and helped ensure that the findings reflect current clinical practice. Furthermore, as this search was restricted to Google Scholar, ProQuest, and PubMed, studies available in other bibliographic databases may not have been identified, which represents a further limitation of this review.

### 2.3 Search strategy

A detailed literature search was performed using Medical Subject Headings (MeSH) terms and keywords related to treatment regimens, cancers, and miR-155. The search was limited to studies published in English. To identify relevant articles, the search for each cancer was subdivided into three treatment categories: (1) chemotherapy, (2) radiotherapy, and (3) drug/multi-drug therapy. Independent keyword combinations were used for each cancer and treatment category, with search terms such as (“Cancer”) AND (“miR-155” OR “miRNA-155” OR “microRNA-155”) AND (“Chemotherapy” OR “Chemosensitivity” OR “Chemoresistance”) AND (“Radiation therapy” OR “Radiosensitivity” OR “Radioresistance”) AND (“Multi-Drug therapy” OR “Combinational therapy” OR “Drug Resistance” OR “Drug sensitivity”) in databases and search engines.

### 2.4 Eligibility criteria

This systematic review aimed to assess miR-155 expression in cancer patients and its association with therapeutic outcomes across different treatment regimens. Patients (male or female) of any age diagnosed with any of the following 15 cancers (lung, breast, colorectal, prostate, stomach, melanoma, hepatocellular carcinoma, thyroid, cervical, esophageal, non-Hodgkin lymphoma, bladder, pancreatic, leukemia, head and neck cancer) were considered for this systematic review. Studies that included patients who received anticancer therapies (radiation, chemotherapy, multi-drug treatments, or combination treatments) and reported survival outcomes according to the therapeutic regimen were also included in the analysis.

The exclusion criteria were as follows: (i) studies that did not assess miR-155 expression in cancer patients undergoing treatment; (ii) research articles without clinical data or assessments; (iii) *in vivo*, *in vitro*, and *in silico* studies; and (iv) book chapters, review articles, unpublished studies, conference proceedings, commentaries, letters to the editor, and retracted studies.

### 2.5 Selection process and data extraction

This systematic review followed the PRISMA 2020 guidelines and assessed the potential role of miR-155 in therapeutic outcomes across studies involving cancer patients. Three reviewers independently extracted the data, which were then consolidated and analyzed. The extracted data included (a) general information about the article (title, author details, and year of publication) and (b) study information (role of miR-155 in the cancers listed above, including clinical outcomes according to the therapeutic strategy and resistance or sensitivity to treatment). Any disagreements regarding data extraction or study selection were resolved, when necessary, through discussion with a fourth reviewer, thereby minimizing selection and extraction bias.

### 2.6 Quality assessment

The data were summarized, and article quality was assessed using the Newcastle-Ottawa Scale (NOS), a validated tool for assessing the methodological quality of observational studies, particularly cohort and case-control studies. NOS evaluates study quality across three domains: selection, comparability, and outcome. This provides a structured assessment of the methodological quality of the included studies. Each study received up to nine stars. Studies scoring fewer than four stars were considered low quality, those scoring four to six stars were considered moderate quality, and those scoring seven or more stars were considered high quality.

## 3 Results

### 3.1 Study selection

The search strategy included all MeSH terms and keywords for individual cancers and treatment types as well as for miR-155. The search syntax was adapted for each database to accommodate differences in indexing and search functionality while preserving the fundamental search principles. As a result, a total of 248,057 records were identified across databases and search engines (ProQuest [n = 77,918], Google Scholar [n = 169,989], and PubMed [n = 150]). Google Scholar returned substantially more records than PubMed because of its broader search coverage and its inclusion of a wider range of scholarly materials. This may also have led to the retrieval of additional studies investigating other miRNAs and their expression in cancer.

Additionally, the extensive search methodology, employing several MeSH terms and keywords, returned a large number of records, including 426 duplicate articles and 245,601 other articles. Most of these were review articles, commentaries, editorials, conference abstracts, or studies of minimal relevance to miR-155 expression and were therefore excluded according to the exclusion criteria. Hence, only studies relevant to the concept of this systematic review were considered for further analysis.

After initial screening, 2,030 articles were assessed by title and then by abstract. Of these, 1,933 articles were excluded during abstract screening based on the predefined inclusion criteria, and 97 studies were identified for full-text retrieval. Of the 97 studies, 78 did not meet the inclusion criteria: 32 lacked validation of miR-155, 26 did not focus on therapy-based analyses in patients, 18 did not evaluate clinical outcomes related to miR-155 expression or had insufficient data, and two focused on simulation or machine learning models without reporting patient data (Figure 1). Thus, 19 studies were considered eligible for inclusion in this systematic review.

**FIGURE 1:**
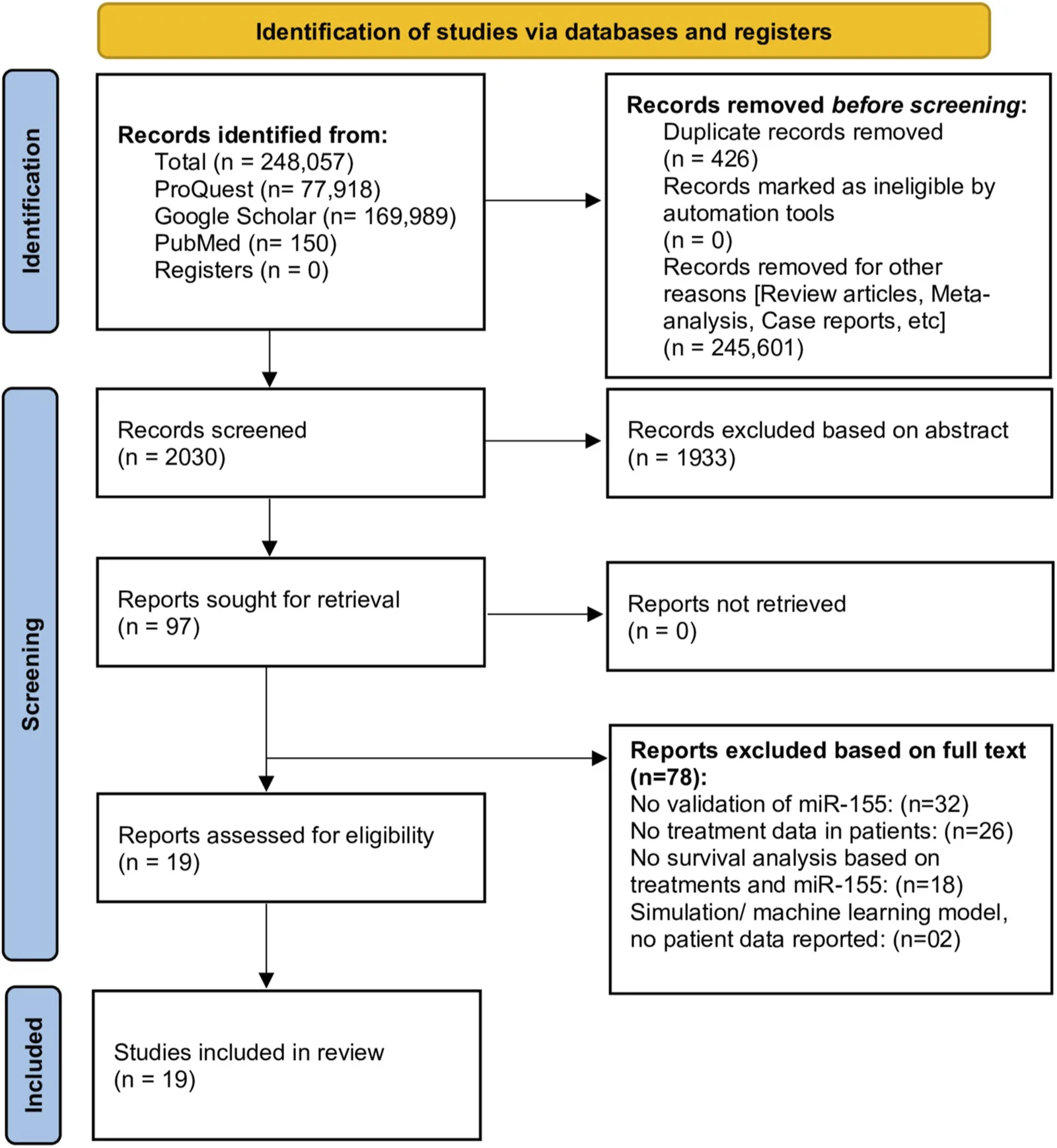
PRISMA flow diagram of the study selection process for this systematic review.

### 3.2 Quality assessment

According to the NOS, most of the 19 studies scored highly in the selection and outcome domains, whereas scores in the comparability domain varied more. Overall, the quality assessment identified no studies with methodological limitations that warranted exclusion from the review (Table 1).

**TABLE 1:** Quality assessment of the included studies using the Newcastle-Ottawa Scale (NOS).

S.No.	Author/Year	Type of study	Selection	Comparability	Outcome	Total score out of 9	Quality of the study
1	Due et al., 2019	Cohort	***	**	***	8/9	High
2	Zheng et al., 2019	Cohort	****	**	**	8/9	High
3	Esplugas et al., 2019	Cohort	***	*	**	6/9	Moderate- high
4	Liu et al., 2019	Cohort	***	-	***	6/9	Moderate- high
5	Zare et al., 2019	Cohort	***	-	**	5/9	Moderate
6	El-Khazragy et al., 2019	Cohort	***	**	***	8/9	High
7	Due et al., 2020	Cohort	***	**	***	8/9	High
8	Papadaki et al., 2020	Cohort	****	**	***	9/9	High
9	Zhang et al., 2020	Cohort	***	**	***	8/9	High
10	Anwar et al., 2020	Cohort	****	**	**	8/9	High
11	Hanafi et al., 2020	Cohort	**	*	***	6/9	Moderate- high
12	Huang et al., 2022	Cohort	***	*	**	6/9	Moderate- high
13	Thomopoulou et al., 2021	Cohort	***	**	***	8/9	High
14	Campennì et al., 2022	Cohort	****	*	**	7/9	High
15	Fawzy et al., 2023	Cohort	***	**	***	8/9	High
16	Tsotridou et al., 2023	Cohort	***	*	**	6/9	Moderate- high
17	Chen et al., 2024	Cohort	***	*	***	7/9	High
18	Darzi et al., 2024	Cohort	***	*	**	6/9	Moderate- high
19	Boyd et al., 2024	Cohort	***	*	**	6/9	Moderate- high

Each study was evaluated using the NOS, with a maximum possible score of nine stars across three categories: Selection (maximum of four stars), Comparability (maximum of two stars), and Outcome (for cohort studies) (maximum of three stars). (*) indicates that the corresponding NOS, criterion was applicable to the study and was included in the quality assessment, (−) indicates that the criterion was not applicable to the study.

11 studies achieved high NOS scores (7–9 out of 9) (Due et al., 2019; Due et al., 2020; El-Khazragy et al., 2019; Zheng et al., 2019; Anwar et al., 2020; Papadaki et al., 2020; Zhang et al., 2020; Thomopoulou et al., 2021; Campennì et al., 2022; Fawzy et al., 2023; Chen et al., 2024). Seven studies achieved moderate-to-high scores (6 out of 9) in the NOS scoring system (Esplugas et al., 2019; Liu et al., 2019; Hanafi et al., 2020; Huang et al., 2022; Tsotridou et al., 2023; Boyd et al., 2024; Darzi et al., 2024). One study scored 5 out of 9 and was considered to have moderate methodological quality (Zare et al., 2019) and it was nonetheless deemed eligible for inclusion because it met the predefined inclusion criteria.

The predominance of high scores indicates that the findings of this review on miR-155 expression in cancer treatment are based largely on studies of high methodological quality.

### 3.3 Study characteristics

Of the 15 cancers considered in this systematic review, eight cancers, namely, diffuse large B-cell lymphoma (DLBCL) and gastric DLBCL, breast cancer, HER2-positive breast cancer, non-muscle-invasive bladder cancer, non-small cell lung cancer (NSCLC), papillary thyroid cancer (PTC), cutaneous melanoma, pancreatic ductal adenocarcinoma (PDAC), and pediatric acute lymphoblastic leukemia (ALL), had studies that assessed miR-155 expression in patients in relation to therapeutic outcomes and met the review’s requirements (Tables 2, 3).

**TABLE 2:** Study characteristics and patient information.

S. No.	Author/Year	Country	Study size	Type of cancer	Sample type for evaluating miR-155 expression	Control patients/Healthy patients (gender)			Cancer patients (gender)			Range/Mean age (In years)	Stage of cancer						
						M	F	NS	M	F	NS		I	II	III	IV	ES	Met	NS
1	Due et al., 2019	Denmark	774 (73 + 701 (GEO database))	DLBCL	Tumor tissue	-	-	-	-	-	774	NS	-	-	-	-	-	-	774
2	Zheng et al., 2019	China	300	DLBCL	Serum and tumor tissue	-	-	100	120	80	-	NS	97		103		-	-	-
3	Esplugas et al., 2019	Spain	136	Breast cancer	Peripheral blood	-	-	-	-	136	-	27-84/56	-	-	-	-	-	-	136
4	Liu et al., 2019	China	123	Breast cancer (HER2-positive)	Serum	-	-	40	-	-	83	31-75/47.6	-	47	36	-	-	-	-
5	Zare et al., 2019	Iran	54	DLBCL	Plasma	3	3	-	23	25	-	-/58.8	15	3	21	9	-	-	-
6	El-Khazragy et al., 2019	Egypt	55	Pediatric ALL	Bone marrow	6	4	-	25	20	-	1.1-15/7.6	-	-	-	-	-	-	45
7	Due et al., 2020	Denmark	774 (73 + 701 (GEO database))	DLBCL	Tumor tissue	-	-	-	447	327	-	17-92/61	-	-	-	-	-	-	774
8	Papadaki et al., 2020	Greece	128	NSCLC	Serum	-	-	19	111	17	-	37-88/65	-	1	4	123	-	112	-
9	Zhang et al., 2020	China	175	Breast cancer (HER2-positive)	Serum exosomes	-	-	-	-	175	-	18-70/-	-	-	-	-	107	68	-
10	Anwar et al., 2020	Indonesia	117	Breast cancer	Plasma	-	15	-	-	102	-	NS	3	45	51	3	-	-	-
11	Hanafi et al., 2020	Indonesia	52	NSCLC	Serum	-	-	-	37	15	-	NS	-	-	3	49	-	-	-
12	Huang et al., 2022	Taiwan	44	Gastric- DLBCL	Tumor tissue	-	-	-	19	25	-	24-100/67	32	11	-	-	-	-	-
13	Thomopoulou et al., 2021	Greece	224	Breast cancer (Triple negative)	Plasma	-	20	-	-	-	204	25-82/57	-	-	-	-	140	64	​
14	Campennì et al., 2022	Italy	69	PTC	Serum	-	-	20	14	35	-	20-75/48	-	-	-	-	-	-	49
15	Fawzy et al., 2023	Egypt	140	Cutaneous melanoma	Tumor tissue	​	​	​	76	-	64	21-90/-	3	76	46	15	-	-	-
16	Tsotridou et al., 2023	Greece	10	Pediatric ALL	Bone marrow	-	-	-	4	6	-	<18/-	-	-	-	-	-	-	10
17	Chen et al., 2024	China	60	Breast cancer	Tumor tissue	-	-	-	-	60	-	43-52/-	37		23		-	≥4	-
18	Darzi et al., 2024	Iran	27	Non-muscle-invasive bladder cancer	Tumor tissue and serum	-	-	-	22	5	-	NS	9	18	-	-	-	-	-
19	Boyd et al., 2024	Italy	DC- 24	PDAC	Plasma	-	-	-	10	14	-	64-74/-	-	-	-	24	-	-	-
			VC- 40	PDAC	Plasma	-	-	-	24	16	-	58-69/-	-	-	15	24	-	-	-

Abbreviations: Acute Lymphoblastic Leukemia (ALL); Discovery Cohort (DC); Diffuse Large B-cell Lymphoma (DLBCL); Early-stage cancer (ES); Female (F); Gene Expression Omnibus (GEO); Male (M); Metastasis (Met); Not Specified (NS); Non-small Cell Lung Cancer (NSCLC); Pancreatic Ductal Adenocarcinoma (PDAC); Papillary Thyroid Cancer (PTC); Validation Cohort (VC); not reported (−).

**TABLE 3:** Patient details according to the treatment received.

S. No	Author/Year	Standardized treatment regimen	Chemotherapy	Radiotherapy	Other/Combinational therapy	Duration of treatment (Days/months/ years)	Follow-up (Days/months/ years)	Response to therapy		Disease status	
								Complete response	Partial response	Stable disease	Progressive disease
1	Due et al., 2019	Vincristine-containing chemotherapy (R-CHOP)	-	-	701 + 68	-	-	-	-	-	-
2	Zheng et al., 2019	R-CHOP-based chemotherapy	-	-	200	-	TC- (6.1–80.8 months) VC- (2.7–58.0 months)	-	-	-	-
3	Esplugas et al., 2019	Radiotherapy, hormone therapy	75	136	131	2–5 months	-	-	-	-	-
4	Liu et al., 2019	NCCT with Taxotere, paraplatin and trastuzumab	83	-	-	4-6 cycles for 21 days	31–36 months	31	31	18	3
5	Zare et al., 2019	R-CHOP	-	-	48	-	-	-	-	-	-
6	El-Khazragy et al., 2019	Induction chemotherapy	45	-	-	-	4 weeks	-	-	-	-
7	Due et al., 2020	Vincristine-containing chemotherapy (R-CHOP)	-	-	701 + 68	-	-	-	-	-	-
8	Papadaki et al., 2020	Platinum-based (cisplatin-based) chemotherapy	128	29	-	-	​	-	33	50	45
9	Zhang et al., 2020	Trastuzumab-based neoadjuvant therapy	ES- 48	-	Met- 65	1 year	ES- 44 months Met- 39 months	Met- 6	Met- 30	Met- 18	Met- 14
10	Anwar et al., 2020	Chemotherapy	40	-	-	-	-	-	-	-	-
11	Hanafi et al., 2020	Non- standardized therapy	-	-	-	-	12 months	-	-	-	-
12	Huang et al., 2022	HPET + chemotherapy	23	-	44	2 weeks	2 months	21		23	
13	Thomopoulou et al., 2021	Adjuvant therapy	Met- 64	-	ES- 140	-	2–182.26 months	-	39	10	15
14	Campennì et al., 2022	Radioiodine treatment with rhTSH stimulation	-	-	49	-	6th and 12th month	-	-	-	-
15	Fawzy et al., 2023	Adjuvant therapy	7	1	140	-	-	68	-	-	45
16	Tsotridou et al., 2023	Induction chemotherapy	10	-	-	-	33 days	-	-	-	-
17	Chen et al., 2024	Post-operative adjuvant chemoradiotherapy	-	-	60	21 days	3 years	-	-	-	-
18	Darzi et al., 2024	BCG immunotherapy	-	-	27	-	20 months	-	-	-	-
19	Boyd et al., 2024	FOLFIRINOX, gemcitabine-nab-paclitaxel	-	-	64	-	-	-	7	9	4
			-	-		-	-	-	16	9	6

Abbreviations: Activated B-cell-like (ABC); *Bacillus* Calmette-Guérin (BCG); Early-stage cancer (ES); Folinic acid, Fluorouracil, Irinotecan and Oxaliplatin (FOLFIRINOX); germinal center B-cell-like (GCB); Gene Expression Omnibus (GEO); *Helicobacter pylori* eradication therapy (HPET); Metastasis (Met); microRNA-155 (miR-155); Neoadjuvant Chemotherapy Combined with Trastuzumab (NCCT); Not Specified (NS); Rituximab, Cyclophosphamide, Doxorubicin, Vincristine, and Prednisone (R-CHOP); Recombinant Human Thyrotropin (rhTSH); Training Cohort (TC); Validation Cohort (VC); not reported (−).

Of the 19 studies, six examined patients with breast cancer (Esplugas et al., 2019; Liu et al., 2019; Anwar et al., 2020; Zhang et al., 2020; Thomopoulou et al., 2021; Chen et al., 2024): Esplugas et al. (2019) examined breast cancer patients with cardiovascular disease (CVD); Liu et al. (2019) and Zhang et al. (2020) examined patients with HER2-positive breast cancer; Thomopoulou et al. (2021) examined patients with triple-negative breast cancer (TNBC); and Anwar et al. (2020) and Chen et al. (2024) examined breast cancer patients without comorbidities. Due et al. (2019), Due et al. (2020) published two studies investigating miR-155 expression in DLBCL using data from the same Gene Expression Omnibus (GEO) datasets (GSE10846 and GSE31312), comprising 701 patients. Although these studies were reported as separate publications, they analyzed overlapping patient populations. Therefore, the 73 retrospective patients and the 701 patients are considered a single underlying cohort rather than two independent patient groups. Huang et al. (2022) conducted a study on gastric DLBCL; Zare et al. (2019) and Zheng et al. (2019) also conducted studies in DLBCL patients without any comorbidities; Fawzy et al. (2023) assessed patients with cutaneous melanoma; Campennì et al. (2022) examined patients with PTC; while Papadaki et al. (2020) and Hanafi et al. (2020) conducted studies on NSCLC; and Tsotridou et al. (2023) and El-Khazragy et al. (2019) conducted studies on pediatric ALL. Darzi et al. (2024) focused on non-muscle-invasive bladder cancer, and Boyd et al. (2024) focused on PDAC.

As mentioned above, two of the 19 studies used the same patient cohort. Accordingly, the patient population from this cohort was counted only once in the overall patient count to avoid duplication. Together, these 19 studies comprised 230 healthy controls, 1,640 patient samples, and data from 701 patients in the GEO datasets (GSE10846 and GSE31312).

miR-155 expression was evaluated in patients using quantitative real-time polymerase chain reaction (qRT-PCR) in tissue, peripheral blood, plasma, plasma exosomes, serum exosomes, and bone marrow samples (Due et al., 2019; Due et al., 2020; Huang et al., 2022; Fawzy et al., 2023; Chen et al., 2024; Esplugas et al., 2019; Liu et al., 2019; Anwar et al., 2020; Hanafi et al., 2020; Papadaki et al., 2020; Thomopoulou et al., 2021; Campennì et al., 2022; Boyd et al., 2024; Darzi et al., 2024; Zare et al., 2019; Zhang et al., 2020; El-Khazragy et al., 2019; Tsotridou et al., 2023). One study evaluated both tissue and blood samples (Zheng et al., 2019) (Table 2).

Table 3 summarizes the number of patients in each study who received various treatments. Some patients received more than one treatment, and five patients in the DLBCL in-house cohort (n = 73), were not treated for unspecified reasons (Due et al., 2019). Thomopoulou et al. (2021) also reported that 236 plasma samples were initially included, of which 32 patient samples were excluded from further evaluation (Thomopoulou et al., 2021). Additionally, a few studies omitted one or two patients from cancer staging (Huang et al., 2022; Boyd et al., 2024).

In the breast cancer studies, patients underwent surgery (tumor removal or radical mastectomy), with treatment regimens primarily involving adjuvant chemotherapy or radiotherapy, neoadjuvant chemotherapy combined with trastuzumab (NCCT) with Taxotere and Paraplatin, and hormone therapy (Esplugas et al., 2019; Liu et al., 2019; Anwar et al., 2020; Zhang et al., 2020; Thomopoulou et al., 2021; Chen et al., 2024).

Four studies focused on DLBCL patients treated with rituximab, cyclophosphamide, doxorubicin, vincristine, and prednisone (R-CHOP) (Due et al., 2019; Due et al., 2020; Zare et al., 2019; Zheng et al., 2019). Two of these four studies divided patients into three groups: (a) a retrospective group (AAU); (b) from the GEO repository: the Lymphoma/Leukemia Molecular Profiling Project R-CHOP (LLMPP R-CHOP) (GSE10846); and (c) the International DLBCL Rituximab-CHOP Consortium MD Anderson Project (IDRC) (GSE31312) (Due et al., 2019; Due et al., 2020). Patients with gastric DLBCL were treated with *Helicobacter pylori* eradication therapy (HPET) combined with chemotherapy when needed to prevent disease progression (Huang et al., 2022).

Patients with NSCLC received platinum-based (cisplatin-based) chemotherapy (Papadaki et al., 2020) or a non-standardized treatment regimen based on disease stage (Hanafi et al., 2020). Patients with pediatric ALL received induction chemotherapy (El-Khazragy et al., 2019; Tsotridou et al., 2023). Patients with cutaneous melanoma received adjuvant therapy, including a polychemotherapy regimen (cisplatin, vinblastine, dacarbazine), dopachrome tautomerase (DCT), interferon, radiotherapy, and sunitinib (Fawzy et al., 2023). Patients with PDAC received folinic acid, fluorouracil, irinotecan, and oxaliplatin (FOLFIRINOX) and gemcitabine-nab-paclitaxel regimens (Boyd et al., 2024). Patients with PTC were treated with radioiodine and recombinant human thyrotropin (rhTSH) stimulation (Campennì et al., 2022) and patients with non-muscle-invasive bladder cancer were treated with *Bacillus* Calmette-Guérin (BCG) immunotherapy (Darzi et al., 2024).

Additionally, 11 studies specified the treatment duration, ranging from 2 weeks to 15 years (El-Khazragy et al., 2019; Esplugas et al., 2019; Liu et al., 2019; Hanafi et al., 2020; Zhang et al., 2020; Thomopoulou et al., 2021; Campennì et al., 2022; Huang et al., 2022; Tsotridou et al., 2023; Chen et al., 2024; Darzi et al., 2024).

Furthermore, eight studies reported patients’ responses to therapy: five reported partial response, five reported stable disease, and six reported progressive disease. In addition, four studies reported complete response, and one study grouped patients with partial response, stable disease, and progressive disease (Table 3).

### 3.4 miR-155 expression and clinical outcomes across cancers

The reported data showed that the association between miR-155 expression and clinical outcomes differed across therapeutic modalities, depending on the specific treatment (Table 4).

**TABLE 4:** miRNA expression and analysis in patients.

S.No.	Author/Year	miR-155 expression in patients after treatment	Sample type/Reported miR-155 expression (Mean ± SEM/Mean ± SD/Median [Q1-Q3]/median expression)/Fold change	Normalization/Technique used for analyzing miR-155	Clinical/biological interpretation of miR-155 in association with treatments	Other miRNAs analyzed
1	Due et al., 2019	Increased	Tumor tissue/-/-	IHC: RNU6B and RNU24/qRT-PCR GEO: RMA/HG-U133 plus 2.0 microarray	Associated with treatment sensitivity and favorable response with improved survival in certain lymphoma subtypes	-
2	Zheng et al., 2019	Increased	Serum and tumor tissue/0.660/-	Serum: miR-39; tissue: RNU6/ qRT-PCR	Associated with shorter PFS. miR-155-specific OS association was not reported	-
3	Esplugas et al., 2019	Increased	Peripheral blood/2.08 ± 0.098/-	snRNA-U6/qRT-PCR	Changes in miR-155 expression were observed following radiotherapy, without a significant association with treatment resistance	miR-146a, miR-221, miR-222
4	Liu et al., 2019	Decreased	Serum/-/-	miR-484/qRT-PCR	Expression was elevated before treatment and decreased after treatment; no significant association with treatment response was observed	miR-10b, miR-21, miR-34a, miR-125b, miR-145, miR-210, miR-373
5	Zare et al., 2019	Increased (exosome)	Exosome (from plasma)/8.401 ± 6.58 vs. 0.13 ± 4.48/- Plasma/0.75 ± 0.66 vs. 1.41 ± 7.55/-	miR-103-5p/qRT-PCR	Associated with relapse/refractory disease and treatment resistance	let-7g, let-7i
6	El-Khazragy et al., 2019	Increased	Bone marrow/-/10-fold (decrease)	SNORD48/qRT-PCR	Associated with poor treatment response, residual disease, and relapse-related clinical characteristics	miR-181a
7	Due et al., 2020	Increased	Tumor tissue/-/-	IHC: RNU6B and RNU24/qRT-PCR GEO: RMA/HG-U133 plus 2.0 microarray	Association with treatment sensitivity, favorable response, and prognosis varied according to disease subtype and treatment modality	-
8	Papadaki et al., 2020	Increased	Serum/-/-	U6 snRNA/qRT-PCR	Associated with poor response and unfavorable prognosis but no significant association with PFS	miR-21, miR-128, miR-181a
9	Zhang et al., 2020	Increased	Serum/-/21.39-fold (increase)	miR-16a/qRT-PCR	Associated with trastuzumab resistance and poorer treatment outcomes and was associated with shorter PFS and EFS	miR-1246, miR-376c, miR-660, miR-99b, miR-665, miR-195, miR-223-3p, miR-30c-5p, miR-6238, miR-222, miR-7040, miR-122, miR-215, miR-885-5p, and miR-3663-3p
10	Anwar et al., 2020	Variable expression	Patients >40 years vs. < 40 years Plasma/(28.92 ± 22 vs. 4.19 ± 2.49)/- patients vs. healthy controls Plasma/(18.49 ± 19 vs. 1.28 ± 0.18)/-	Sp6 spike-in and miR-16/qRT-PCR	Expression was elevated before treatment and decreased after treatment and was associated with longer PFS	-
11	Hanafi et al., 2020	Increased	Serum/-/-	miR-16/qRT-PCR	Associated with poor prognosis and poor survival outcomes, particularly in adenocarcinoma and EGFR mutation-positive patients	miR-34, miR-148, miR-222
12	Huang et al., 2022	Increased	Tumor tissue/-10.4 ± 0.2 vs. −8.6 ± 0.8/4-fold (increase)	U6/stem-loop qRT-PCR	Associated with resistance to HPET; however, no significant difference in OS or PFS was observed between treatment-response groups	miR-1203, miR-200a, b, c, miR-22, miR-31, miR-141, miR-192, miR-194
13	Thomopoulou et al., 2021	Decreased	Plasma/-/-	U6 snRNA/qRT-PCR	Lower expression was associated with shorter DFS	miR-10b, miR-19a, miR-20a, miR-126
14	Campennì et al., 2022	Decreased	Serum/3.11 ± 0.66 vs. 1.20 ± 0.77/-	RNU6/qRT-PCR	A reduction in miR-155 levels from baseline was observed in patients responding to therapy	miR-221, miR-222, miR-375, miR-146b
15	Fawzy et al., 2023	Increased	Tumor tissue/-/-	RNU6B/qRT-PCR	Higher expression was associated with a higher mitotic rate in superficial melanoma	-
16	Tsotridou et al., 2023	Variable expression	Bone marrow/-/-	Let-7a-5p/qRT-PCR	Association with early treatment response; expression differed between good and poor responders	let-7c-5p, miR-106b-5p, miR-26a-5p, miR-191-5p, miR-30b-5p, miR-31-5p, miR-125b-5p, miR-150-5p, miR-999-5p
17	Chen et al., 2024	Increased	Tumor tissue/0.86 ± 0.22 vs. 0.71 ± 0.23/-	U6/qRT-PCR	Associated with poor prognosis and an aggressive disease phenotype, without a specific treatment-response association	miR-182, miR-217
18	Darzi et al., 2024	Increased	Tissue/0.57-2.08/1.3-fold (increase)	RNU6-1/qRT-PCR	A slight increase in miR-155-5p was observed after treatment, with limited evidence for its association with therapeutic response	-
19	Boyd et al., 2024	No significant difference	Plasma/-/-	miR-16/qRT-PCR	Association between miR-155 expression and treatment response was not significant	miR-127, miR-200, miR-379

Abbreviations: *Bacillus* Calmette-Guérin (BCG); Disease-free Survival (DFS); Event-free survival (EFS); Epidermal Growth Factor Receptor (EGFR); Gene Expression Omnibus (GEO); Affymetrix Human Genome U133 Plus 2.0 Array (HG-U133, Plus 2.0); *Helicobacter pylori* eradication therapy (HPET); In-House Cohort (IHC); microRNA-155 (miR-155); Overall Survival (OS); Progression-free Survival (PFS); first quartile (Q1); third quartile (Q3); Quantitative Real-Time Polymerase Chain Reaction (qRT-PCR); Robust Multi-array Average (RMA); standard deviation (SD); standard error of the mean (SEM). miR-155 expression and fold-change values are reported as presented in the respective published studies, using the original measurement format and units where provided. Mean ± SEM, Mean ± SD, Median [Q1-Q3], and median expression values represent the summary measures reported by the authors. Fold change values are likewise reported directly from the respective studies. No expression values or fold-change estimates were statistically calculated, recalculated, or transformed in the present review.

Of the 19 included studies, four reported elevated miR-155 expression in treatment-resistant patients (El-Khazragy et al., 2019; Zare et al., 2019; Zhang et al., 2020; Huang et al., 2022), and two (Due et al., 2019; Due et al., 2020) reported that increased miR-155 expression following treatment was associated with improved patient survival. In addition, a few of the included studies analyzed various other miRNAs including miR-10b, miR-19a, miR-20a, miR-21, miR-22, miR-26a-5p, miR-30b-5p, miR-30c-5p, miR-31, miR-31-5p, miR-34, miR-34a, miR-99b, miR-106b-5p, miR-122, miR-125b, miR-125b-5p, miR-126, miR-127, miR-128, miR-141, miR-145, miR-146a, miR-146b, miR-148, miR-150-5p, miR-181a, miR-182, miR-191-5p, miR-192, miR-194, miR-195, miR-200, miR-200a, miR-200b, miR-200c, miR-210, miR-215, miR-217, miR-221, miR-222, miR-223-3p, miR-373, miR-375, miR-376c, miR-379, miR-660, miR-665, miR-999-5p, miR-1203, miR-1246, miR-7040, miR-6238, miR-885-5p, miR-3663-3p, let-7g, let-7i and let-7c-5p along with miR-155 in patient samples (Esplugas et al., 2019; Liu et al., 2019; Papadaki et al., 2020; Zhang et al., 2020; Tsotridou et al., 2023; Chen et al., 2024).

Overall, miR-155 expression showed variable associations with treatment outcomes across the included studies, and these differences varied by cancer type, treatment modality, and clinical context.

#### 3.4.1 miR-155 expression and treatment resistance

Across the included studies, elevated miR-155 expression was more frequently associated with treatment resistance than with response. Treatment resistance was defined according to the original study’s clinical designation and according to the reported evidence of refractory/non-responsive/progressive disease following treatment. Four studies reported resistance-associated elevation of miR-155 in distinct clinical settings, including induction chemotherapy in pediatric ALL, trastuzumab-containing therapy in HER2-positive breast cancer, R-CHOP treatment in DLBCL, and HPET treatment in gastric DLBCL (El-Khazragy et al., 2019; Zare et al., 2019; Zhang et al., 2020; Huang et al., 2022).

The resistance-associated findings were observed across different biological specimens rather than being restricted to a single specimen type, including circulating serum- or blood-derived miR-155, exosomal miR-155, and tumor-tissue miR-155. Positive resistance-associated findings were reported in four studies: one on chemotherapy in bone marrow samples (El-Khazragy et al., 2019), one on targeted therapy in serum samples (Zhang et al., 2020), one on immunochemotherapy in plasma-derived exosomes (Zare et al., 2019), and one on HPET with combination therapy in tissue samples (Huang et al., 2022). In contrast, an additional analysis of immunochemotherapy in plasma samples yielded null findings (Zare et al., 2019). No resistance-associated findings were reported for radiotherapy or immunotherapy. Thus, the findings did not indicate a uniform association within a given specimen category. Notably, in DLBCL, exosomal miR-155 differentiated refractory or relapsed patients from treatment-responsive patients, whereas this association was not observed with plasma miR-155 (Zare et al., 2019). This suggests that the type of biological specimen used to measure miR-155 may influence its ability to reflect treatment resistance.

The association between miR-155 and treatment outcome also varied according to therapeutic modality across the included studies. Resistance-associated findings were reported mainly in chemotherapy and targeted-therapy settings, whereas evidence from other therapies was less consistent. The available studies therefore do not support a universal resistance-associated role for miR-155 across all therapeutic modalities. Instead, the strength of the association between miR-155 expression and patient outcomes appears to depend on cancer type, treatment regimen, and the type and source of the sample in which miR-155 expression is assessed.

#### 3.4.2 miR-155 expression and treatment response and sensitivity

The relationship between miR-155 expression and treatment response or sensitivity was heterogeneous across the included studies. Favorable associations were reported in hematological settings, specifically in germinal center B-cell-like (GCB)-DLBCL treated with R-CHOP and pediatric ALL during early prednisone response, whereas several solid-tumor studies reported non-significant or context-dependent associations (Due et al., 2019; Due et al., 2020; Tsotridou et al., 2023). Conversely, elevated miR-155 was associated with treatment resistance and poor treatment response in several other clinical settings, including pediatric ALL, HER2-positive breast cancer, and gastric DLBCL (El-Khazragy et al., 2019; Zhang et al., 2020; Huang et al., 2022). Contrasting findings in DLBCL appear to be influenced by molecular subtype and clinical context. Higher tissue miR-155 expression was associated with favorable response to R-CHOP in GCB-DLBCL, whereas no comparable therapeutic stratification was observed in activated B-cell-like (ABC)-DLBCL (Due et al., 2019; Due et al., 2020). In contrast, elevated exosomal miR-155 was associated with refractory or relapsed disease in DLBCL (Zare et al., 2019).

Differences between the 12 studies on solid tumors and the seven on hematological malignancies may reflect underlying differences in disease-specific biological characteristics and treatment context; however, the available clinical evidence is insufficient to determine the independent contribution of these factors to the observed variation in miR-155 expression and treatment outcomes. In solid tumors, circulating miR-155 generally showed weak, non-significant, or context-dependent associations with treatment response. Serum miR-155 did not independently distinguish responders from non-responders in HER2-positive breast cancer receiving neoadjuvant chemotherapy (Liu et al., 2019), while plasma miR-155 did not significantly distinguish treatment response in patients with advanced PDAC receiving different chemotherapy regimens (Boyd et al., 2024). Similarly, treatment-associated changes in circulating miR-155 in radiotherapy-treated breast cancer and in BCG-treated bladder cancer did not show a clear association with treatment response or resistance (Esplugas et al., 2019; Darzi et al., 2024). Additionally, sample source may contribute to the observed differences, but it does not fully account for the heterogeneity. In DLBCL, tissue miR-155 was associated with favorable response in GCB-DLBCL, whereas exosomal miR-155 was associated with refractory or relapsed disease (Due et al., 2019; Due et al., 2020; Zare et al., 2019). However, contrasting findings were also observed within the same cancer and broadly similar treatment setting in pediatric ALL, where elevated miR-155-5p was associated with favorable early prednisone response in one study, whereas another study reported an association between miR-155 and suboptimal treatment response (El-Khazragy et al., 2019; Tsotridou et al., 2023). These observations suggest that cancer subtype and clinical context may contribute substantially to the direction of the reported association, potentially more strongly than specimen type alone.

Taken together, the findings suggest that the association of miR-155 with treatment outcomes is influenced by disease-specific biological context, including cancer subtype, treatment setting, and specimen type. Although subtype-specific variation was evident in both hematological and solid malignancies, it was most pronounced in DLBCL studies, whereas breast cancer studies showed greater consistency even when distinct clinical subtypes were included (HER2-positive breast cancer and TNBC). This pattern suggests that disease subtype may influence miR-155 expression and its treatment-related associations across malignancies, rather than reflecting a uniform effect of miR-155 across cancers.

#### 3.4.3 Survival outcome-based analysis of miR-155 expression

Among the included studies, favorable survival associations were reported by four studies (Due et al., 2019; Due et al., 2020; Anwar et al., 2020; Thomopoulou et al., 2021), whereas adverse survival associations were reported by four other studies (Zheng et al., 2019; Papadaki et al., 2020; Zhang et al., 2020; Chen et al., 2024). One study reported endpoint-dependent findings, with associations between higher miR-155 expression and survival varying across endpoints and patient subgroups (Hanafi et al., 2020). Huang et al. (2022) reported a null survival association with no statistically significant difference in overall survival (OS) or progression-free survival (PFS) between treatment-response groups. Nine studies did not perform a miR-155-specific survival analysis (El-Khazragy et al., 2019; Esplugas et al., 2019; Liu et al., 2019; Zare et al., 2019; Campennì et al., 2022; Fawzy et al., 2023; Tsotridou et al., 2023; Boyd et al., 2024; Darzi et al., 2024). Thus, the survival findings comprised statistically significant favorable associations, statistically significant adverse associations, mixed or endpoint-dependent findings, null or non-significant findings, and studies without a miR-155-specific survival analysis. These findings indicate that the association between miR-155 expression and survival was not uniform across the included clinical settings.

The survival outcomes of these studies demonstrated a heterogeneous relationship between miR-155 expression and clinical prognosis across cancer types and treatment settings. Statistically significant adverse associations were reported in selected DLBCL, NSCLC, and breast cancer cohorts, particularly in chemotherapy- and targeted-therapy settings (Zheng et al., 2019; Zhang et al., 2020; Chen et al., 2024). Higher miR-155 expression was generally associated with shorter PFS, OS, or event-free survival (EFS) in these settings, although this prognostic significance was not consistently maintained and the statistical significance of these associations varied across studies (Zheng et al., 2019; Hanafi et al., 2020; Papadaki et al., 2020; Zhang et al., 2020; Chen et al., 2024). Similarly, in gastric DLBCL treated with HPET, higher miR-155 expression was associated with treatment resistance, but the corresponding differences in 5-year OS and PFS between resistant and sensitive groups were not statistically significant. This suggests that the association between miR-155 expression and treatment resistance may not translate into differences in survival outcomes (Huang et al., 2022).

In contrast, favorable survival associations were identified in selected disease subtypes and treatment contexts. Higher miR-155 expression was associated with better OS and PFS in GCB-DLBCL treated with R-CHOP but not in ABC-DLBCL, while higher expression was also associated with longer disease-free survival (DFS), OS, or PFS in selected breast cancer cohorts, including TNBC and patients receiving adjuvant therapy (Due et al., 2019; Due et al., 2020; Anwar et al., 2020; Thomopoulou et al., 2021).

Hence, differences in survival outcomes were particularly evident in DLBCL and breast cancer, for which both favorable and unfavorable associations with miR-155 expression were reported across different disease subtypes and treatment settings. In DLBCL, the association varied by subtype, with favorable survival outcomes reported in GCB-DLBCL but not consistently in ABC-DLBCL, while other DLBCL cohorts showed adverse or non-significant survival patterns. Similarly, breast cancer studies reported both favorable and unfavorable survival associations across HER2-positive breast cancer, TNBC, and different treatment settings. These contrasting findings may reflect differences in tumor biology, molecular subtype, treatment regimen, patient characteristics, and specimen type, rather than a uniform prognostic effect of miR-155 across cancers. The limited number of studies within individual cancer subtypes and treatment settings further restricts the ability to determine which of these factors contributes most to the observed differences.

Differences in survival endpoints also contributed to the heterogeneity. Associations were reported with PFS, EFS, DFS, and OS, and the same study did not necessarily demonstrate the same relationship across all endpoints. For example, elevated exosomal miR-155 in HER2-positive breast cancer was associated with poorer EFS or PFS but not significantly different OS (Zhang et al., 2020). Similarly, NSCLC studies showed associations with OS that varied in statistical significance and depended on patient subgroups. In NSCLC patients receiving first-line platinum-based chemotherapy, higher circulating miR-155 was associated with poorer OS, although it was not significantly associated with treatment response or PFS (Papadaki et al., 2020), whereas in serum samples from NSCLC patients, an adverse prognostic association was observed particularly in the adenocarcinoma and EGFR mutation-positive patients (Hanafi et al., 2020). Huang et al. (2022) further illustrated this distinction: treatment resistance was associated with higher miR-155 expression, whereas the corresponding differences in OS and PFS between treatment-response groups were not statistically significant. These findings indicate that treatment resistance and survival associations should be considered distinct clinical outcomes and should not be assumed to have the same relationship with miR-155 expression.

Overall, the survival evidence included statistically significant favorable associations, statistically significant adverse associations, mixed or endpoint-dependent findings, null or non-significant findings, and studies without a miR-155-specific survival analysis. Favorable associations were predominantly reported in specific clinical and molecular contexts, particularly in GCB-DLBCL and TNBC, whereas unfavorable associations were more frequently reported in selected chemotherapy and targeted-therapy settings. Importantly, as Huang et al. (2022) reported, treatment resistance does not necessarily correspond to a statistically significant adverse survival association.

### 3.5 Clinical heterogeneity in association of miR-155 expression with treatment outcomes

Considerable clinical heterogeneity was observed among the 19 included studies in cancer type, disease subtype, patient characteristics, treatment modality, biological specimen, timing of sample collection, endpoint definition, and follow-up duration. These differences complicate direct comparison of miR-155 expression levels and their associations with therapeutic outcomes.

#### 3.5.1 Cancer type and disease subtype

As mentioned, the 19 included studies encompassed eight cancer types, and substantial heterogeneity was observed in the relationship between miR-155 expression and treatment-related outcomes across these malignancies. This variation may reflect differences in the underlying tumor biology and molecular characteristics of each cancer type and disease subtype. miR-155 expression is generally influenced by the tumor’s cellular and molecular environment, and its biological relevance may therefore differ between malignancies. Differences in tumor subtype, disease stage, cellular composition, and the pathways involved in treatment response may influence both miR-155 expression levels and their clinical significance.

This was particularly evident in DLBCL, where the association differed between molecular subtypes, with favorable survival associations reported in GCB-DLBCL but not consistently in ABC-DLBCL. Similarly, breast cancer studies included distinct clinical and molecular subgroups, including HER2-positive breast cancer and TNBC, and reported both favorable and unfavorable associations between miR-155 expression and treatment outcomes. In NSCLC and pediatric ALL, miR-155 was evaluated primarily in relation to treatment resistance or unfavorable response, whereas studies of cutaneous melanoma, PTC, non-muscle-invasive bladder cancer, and PDAC reflected different tumor biology and treatment contexts. Thus, cancer type and subtype may influence the biological context in which miR-155 is expressed and whether elevated miR-155 is associated with treatment resistance, treatment response, or survival. These findings support a context-dependent role for miR-155 rather than a uniform role across malignancies.

#### 3.5.2 Treatment modality and treatment context

Across the 19 studies, patients received different treatments, including chemotherapy, platinum-based therapy, immunochemotherapy, targeted therapy, and combination regimens, reflecting substantial variation in treatment settings. In some of these studies, elevated miR-155 expression was associated with unfavorable treatment-related outcomes and resistance, including platinum-based treatment in NSCLC, trastuzumab-containing treatment in HER2-positive breast cancer, and R-CHOP and HPET-related treatment settings in DLBCL and gastric DLBCL, respectively (Zare et al., 2019; Papadaki et al., 2020; Zhang et al., 2020; Huang et al., 2022). Conversely, some DLBCL investigations reported associations between elevated miR-155 expression and favorable treatment-related outcomes (Due et al., 2019; Due et al., 2020). These contrasting findings suggest that the clinical significance of miR-155 may depend on the therapeutic context rather than being consistent across treatments. Furthermore, differences in treatment mechanisms may contribute to the observed variation. In particular, the same miR-155 expression pattern may have different clinical implications depending on the tumor’s biological characteristics and the prescribed treatment. Therefore, elevated miR-155 should not be interpreted uniformly as a marker of either treatment resistance or sensitivity. Instead, its relationship with treatment outcome appears to be context-dependent, which may explain the contrasting associations observed across the included studies.

#### 3.5.3 Biological specimen

Substantial variation was also observed in the biological sources used to assess miR-155. The included studies analyzed tumor tissue, peripheral blood, serum, exosomal samples, and bone marrow. Tissue-based analyses reflect miR-155 expression within the tumor or local tissue environment, whereas circulating and exosomal measurements capture miR-155 in systemic or extracellular compartments. This distinction was particularly relevant in breast cancer and DLBCL, where clinical cohorts used different specimen types. Therefore, associations observed in tissue-based and circulating samples should be interpreted cautiously, as differences in specimen source may contribute to heterogeneity in reported findings. In cutaneous melanoma, tissue miR-155 expression also varied according to clinicopathological characteristics, with higher expression observed in superficial spreading melanoma and in samples with a high mitotic rate (Fawzy et al., 2023).

#### 3.5.4 Timing of sample collection and endpoint assessment

The timing of specimen collection relative to treatment also differed among studies. Samples were obtained at different clinical time points, including before, during, and/or after treatment, depending on the study design. Because miR-155 expression may vary with treatment exposure and disease status, measurements from different time points may not reflect the same biological or clinical state. This time-related heterogeneity therefore limits direct comparison of expression levels between studies.

The clinical endpoints used to characterize treatment-related outcomes were also heterogeneous. Studies assessed treatment resistance, treatment response or sensitivity, early treatment response, minimal residual disease-related outcomes, PFS, OS, and other prognostic or clinicopathological outcomes. Importantly, these endpoints do not represent equivalent clinical outcomes. For example, an association between miR-155 expression and an early treatment-response measure cannot be directly equated with an association with long-term survival or treatment resistance. This variation in endpoint definitions complicates the interpretation of the role of miR-155 in cancer progression and treatment outcomes.

#### 3.5.5 Follow-up duration and clinical assessment period

Follow-up duration and the period over which studies assessed clinical outcomes also varied. Differences in follow-up periods may influence the detection of progression, relapse, survival events, or sustained treatment response. Accordingly, studies with short-term treatment-response outcomes and those evaluating longer-term survival outcomes provide different levels of clinical information and should not be interpreted interchangeably. An additional methodological source of heterogeneity was the use of both primary clinical cohorts and secondary analyses of publicly available datasets. For example, Due et al. (2019), Due et al. (2020) investigated miR-155 in a clinical cohort of 73 patients with DLBCL and subsequently examined associations with treatment outcomes using publicly available GEO datasets, including GSE10846 and GSE31312. These analyses therefore incorporated both directly collected patient-level data and secondary molecular and clinical data, which should be distinguished when interpreting the evidence.

Conclusively, clinical heterogeneity across studies likely influenced the observed association between miR-155 expression and treatment outcomes, reflecting biological processes that differ between cancer types and molecular subtypes. Differences in cancer subtype and treatment regimen produced distinct associations, while tissue, blood/serum, and exosomal samples represent different biological specimen and therefore showed different expression patterns. Similarly, differences in sampling time resulted in variation between pre- and post-treatment miR-155 levels. The cross-sectional design of several studies also limited the ability to determine whether altered miR-155 expression preceded or followed treatment response or resistance.

## 4 Discussion

This systematic review evaluated the available clinical evidence on miR-155 expression and its reported association with therapeutic outcomes across cancer types. Overall, the findings demonstrated considerable variability in the clinical association of miR-155. Although high miR-155 expression was reported in patients with unfavorable treatment-related outcomes, this pattern was not consistently observed across all cancers, and the association between miR-155 expression and therapeutic outcomes was context-dependent rather than uniform across cancer types.

### 4.1 Quality assessment and risk of bias

This review predominantly comprises observational studies examining the relationship between miR-155 expression and treatment resistance or clinical outcomes, rather than studies focused on developing or validating prognostic models. Conclusions about the predictive or prognostic value of miR-155 would require biomarker-specific evaluation and validation using appropriate assessment frameworks. Accordingly, statistically significant associations in individual studies cannot establish the clinical significance of miR-155. Hence, the NOS was deemed suitable for evaluating the methodological rigor of the included observational studies. The methodological quality of the included studies was generally favorable, with 11 studies classified as high quality and eight as moderate-to-high quality; no study was classified as low quality. However, scores differed in the comparability domain. Nine studies received the maximum two comparability stars, eight received one star, and two studies received no comparability stars. This indicates that comparability between study groups and adjustment for potential confounding factors were not consistent across the included studies. Limited adjustment for relevant clinical and pathological characteristics may have influenced the associations between miR-155 expression and treatment outcomes. Therefore, residual confounding remains a potential concern, particularly in studies with limited comparability, and may have contributed to the heterogeneity and uncertainty observed across the evidence base. Consequently, observed associations between miR-155 expression and therapeutic outcomes may partly reflect differences in patient characteristics, disease-related factors, or treatment-related variables rather than an independent association with miR-155. Furthermore, this review did not aim to propose miR-155 as a therapeutic target, but rather to assess the available clinical evidence on its expression and reported associations in therapeutic settings, particularly in treatment response and resistance. Accordingly, the observed associations should be interpreted as evidence of a potential clinical relationship rather than definitive evidence supporting miR-155, given the limited number of studies identified owing to the restricted databases and timeframe.

### 4.2 Methodological variations

The methodological quality of the included studies also varied, with differences in study design, sample size, outcome definitions, and sampling methods. Although the included studies provided clinical evidence on miR-155 and treatment outcomes, several had relatively small patient populations. Six of the 19 included studies (31.6%) enrolled 55 or fewer participants, including one study with only 10 patients. Such small sample sizes may reduce statistical power and produce imprecise and unstable effect estimates. In addition, small cohorts may be more susceptible to random variation and may increase the risk of false-positive findings, particularly when multiple clinical or molecular outcomes are assessed. Therefore, associations reported in small studies should be interpreted cautiously and considered preliminary until replicated in larger, independent patient cohorts. Differences in specimen type, collection procedures, processing, and analytical methods may also contribute to variation in measured expression and limit direct comparison between studies. In addition, variations in study design, including prospective and retrospective approaches and analyses based on directly collected clinical samples versus publicly available datasets, may have introduced variability in patient selection and in the assessment of miR-155-related outcomes. Moreover, the included studies used different criteria and follow-up periods to measure treatment resistance, response, sensitivity, recurrence, PFS, OS, DFS, and EFS, among other clinical outcomes. Notably, favorable associations and non-significant findings were reported in a few clinical settings, highlighting the context-dependent nature of miR-155 expression during treatment. An additional source of heterogeneity was the inconsistent reporting of mature miRNA isoforms. 16 of the 19 included studies referred simply to “miR-155” without specifying the mature strand, whereas only three studies explicitly evaluated miR-155-5p (Fawzy et al., 2023; Tsotridou et al., 2023; Darzi et al., 2024). Because mature miRNA strands may differ in sequence, target interactions, and biological effects, this inconsistency may limit direct comparisons across studies. Taken together, these methodological and clinical differences reduce comparability and warrant caution when interpreting miR-155 across clinical settings.

### 4.3 Comparison with previous evidence

Previous studies have reported that miR-155 is frequently dysregulated in cancers following treatment and is associated with survival outcomes; however, these associations have been inconsistent across cancer types (Abdul-Rahman et al., 2026; Tili et al., 2026). In this review, breast cancer was the most frequently studied cancer within the 5-year timeframe (Esplugas et al., 2019; Liu et al., 2019; Anwar et al., 2020; Zhang et al., 2020; Thomopoulou et al., 2021; Chen et al., 2024). Some of these studies reported that elevated miR-155 expression was associated with treatment resistance, poor prognosis, and shorter DFS, PFS, and EFS, particularly in patients receiving radiotherapy or chemotherapy- or trastuzumab-based treatments (Esplugas et al., 2019; Zhang et al., 2020; Chen et al., 2024). In contrast, data from DLBCL illustrate the biological complexity of miR-155, where increased circulating and exosomal miR-155 expression was associated with treatment resistance, refractory disease, and reduced PFS, but its prognostic importance varied by molecular subtype (Zare et al., 2019). Conversely, analyses of patient-derived samples and databases showed contrasting results, with increased expression associated with enhanced treatment sensitivity (Due et al., 2019; Due et al., 2020). These findings suggest that the clinical relevance of miR-155 in DLBCL is influenced by disease heterogeneity. Evidence from NSCLC and pediatric ALL was similarly heterogeneous, with elevated miR-155 associated with treatment resistance or unfavorable outcomes in some studies, though these associations varied by treatment modality and clinical context (El-Khazragy et al., 2019; Hanafi et al., 2020; Papadaki et al., 2020; Tsotridou et al., 2023). Similarly, the limited evidence from PDAC, melanoma, and non-muscle-invasive bladder cancer does not support miR-155 as a predictor of treatment response or survival, despite dysregulated expression (Fawzy et al., 2023; Boyd et al., 2024; Darzi et al., 2024). However, in non-muscle-invasive bladder cancer, the association of miR-155 with oxidative stress markers may indicate a role in the biological response to therapy rather than direct prediction of clinical outcomes (Darzi et al., 2024), while changes in circulating miR-155 following radioiodine treatment in PTC suggest potential utility for treatment monitoring rather than prediction (Campennì et al., 2022). Studies have also shown that combining miR-155 with other miRNAs improves prediction which suggests that multi-marker approaches warrant further investigation rather than demonstrating that miR-155 alone has sufficient predictive accuracy (Thomopoulou et al., 2021; Chen et al., 2024). In addition, non-significant findings, including those reported by Liu et al. (2019) and Boyd et al. (2024), further emphasize the variable clinical value of miR-155 across treatment settings. Although mechanistic studies were outside the scope of this review, experimental evidence suggests that miR-155 may contribute to chemoresistance by regulating apoptosis, immune-related pathways, and epithelial-mesenchymal transition (Zang et al., 2012; Dhuri et al., 2022). These findings provide biological context for the treatment-related associations observed in some included clinical studies, but they do not constitute direct clinical evidence. Similarly, previous translational studies have explored miR-155 as a diagnostic or therapeutic target across different malignancies, while broader reviews have emphasized the potential of miRNA- and ncRNA-based approaches (Shao et al., 2019; Tang et al., 2019; Wu et al., 2023; Grillone et al., 2024). However, ongoing and discontinued clinical investigations indicate that translating these findings into clinical applications remains challenging because expression patterns vary across cancer types and therapeutic efficacy is inconsistent (Liu et al., 2018; Tang et al., 2019; Qian et al., 2024).

### 4.4 Concluding remarks

Of the 19 included studies, 11 (57.9%) were classified as high methodological quality and eight (42.1%) as moderate quality, with no studies classified as low quality. However, scores in the comparability domain varied, with nine studies (47.4%) receiving two stars, eight (42.1%) receiving one star, and two (10.5%) receiving no stars, indicating variable adjustment for potential confounding factors. In addition, substantial heterogeneity was observed across the clinical endpoints, with ten studies (47.4%) examining survival or prognostic outcomes, with some studies addressing more than one endpoint. As discussed above, differences in cancer type, treatment modality, biological specimen, sampling time, and outcome definitions further limited direct comparison across studies. The predominantly observational evidence and the relatively small sample sizes of several cohorts also reduced confidence in the consistency and generalizability of the observed associations. Overall, although the methodological quality of the included evidence was generally favorable, substantial clinical and methodological heterogeneity limits confidence in the association between miR-155 expression and treatment-related outcomes.

Regarding the independence and strength of the reported findings, most treatment-related associations were based on single studies rather than repeated evaluation in independent patient cohorts. The two publications by Due et al. (2019), Due et al. (2020) were based on the same underlying DLBCL cohort and dataset and are therefore not considered independent replication of the reported findings. Additionally, as mentioned above, several associations were based on cohorts of 55 or fewer participants and therefore represent preliminary analyses that require confirmation in larger independent cohorts. Furthermore, even in studies with more than 55 patients overall, some analyses were based on relatively small individual cohorts. For example, one study included 64 patients overall, but the discovery and validation cohorts comprised 24 and 40 patients, respectively. Although the association was evaluated across both cohorts, the relatively small size of each individual cohort should be considered when interpreting the findings (Boyd et al., 2024).

Overall, confidence in the evidence linking miR-155 expression with therapeutic outcomes remains limited. Accordingly, findings supported by multiple independent studies may provide more reliable evidence, whereas associations derived from single or small studies should be regarded as preliminary. Larger, well-designed prospective studies with standardized sampling, clearly defined therapeutic endpoints, and appropriate adjustment for potential confounding are required to establish the clinical utility of miR-155 as a predictive or prognostic biomarker.

## 5 Future translational directions

This systematic review indicates that the clinical interpretation of miR-155 remains largely dependent on cancer type, disease characteristics, treatment context, biological specimen, sampling time, and outcome definition. Given these sources of heterogeneity, future research should prioritize clinical validation and methodological standardization before considering miR-155-directed therapeutic applications.

Additionally, this systematic review did not identify or include clinical efficacy evidence evaluating miR-155-directed therapeutic interventions. The available clinical evidence is currently insufficient to establish miR-155 as a universally applicable predictive or prognostic biomarker across cancers. Future studies should therefore use well-defined and clinically relevant patient cohorts, preferably through prospective, multicenter studies with larger sample sizes, to determine whether associations observed in individual cancer types and treatment settings can be independently validated. Researchers should also ensure methodological uniformity with respect to predefined patient characteristics, treatment regimens, disease subtypes, and clinically meaningful endpoints.

Standardization of miR-155 assessment is also needed to improve comparability between studies. Future investigations should clearly define the biological specimen used for measurement and the procedures for sample collection, processing, RNA extraction, normalization, and miRNA quantification. Moreover, reporting whether total miR-155 or a specific mature form, such as miR-155-5p, is measured would also improve interpretation. In addition, studies should clearly state the timing of sample collection relative to treatment initiation, treatment exposure, response assessment, and follow-up. Sequential sampling may also help determine whether changes in miR-155 during treatment are associated with treatment response, resistance, recurrence, or disease progression.

As mentioned earlier, consistent clinical endpoint definitions and follow-up periods are also required. Future studies should use standardized definitions of treatment response, treatment resistance, progression, recurrence, PFS, and OS, with adequately defined follow-up intervals. This would enable more meaningful comparisons between studies and facilitate quantitative synthesis where appropriate. The studies reviewed also highlight the need to determine whether the clinical significance of miR-155 is cancer- and treatment-specific rather than universal. Future research should therefore prioritize validation within clearly defined cancer subtypes and therapeutic settings rather than assuming that an association identified in one clinical context applies across malignancies. Independent validation cohorts and external validation using standardized datasets would further strengthen the evidence base.

Furthermore, mechanistic, preclinical, and simulation-based studies, which were excluded from this review, have investigated the potential effects of miR-155 modulation and combinations with existing therapies (Bayraktar and Van Roosbroeck, 2018; Dhuri et al., 2022; Dogra et al., 2024). These studies may provide hypotheses for subsequent investigation. Likewise, any future evaluation of anti-miR-155 in clinical settings should proceed through appropriately designed preclinical studies followed by carefully controlled early-phase clinical investigations to establish safety, feasibility, and clinical efficacy.

Overall, further research is needed to determine whether miR-155 is reliably associated with patient outcomes, and to establish the cancer types, treatment settings, and standardized measurement conditions in which its clinical utility may be most relevant. Larger prospective multicenter cohorts, standardized miRNA assessment procedures, sequential assessment, uniform clinical endpoints, and independent validation are needed to confirm its clinical utility.

## 6 Limitations

This systematic review focused on miR-155, evaluating its expression and its association with therapeutic outcomes across 15 cancer types. Based on the inclusion criteria and the clinical relevance of this study, only eight cancers and a limited number of studies were included. Another limitation was that only research articles published between 2019 and 2024 were considered, as specified in the eligibility criteria. Additionally, the use of a limited number of databases may have restricted the identification of relevant studies. Although these databases contain a wide range of research publications, studies indexed in other bibliographic sources may not have been identified. Hence, the inferences drawn in this review regarding miR-155 expression may not fully represent all cancer types and treatment approaches. Consequently, including studies indexed in other databases may strengthen the conclusions of this systematic review.

The quality assessment indicated that most studies were of moderate to high quality, suggesting that the overall findings were supported by studies with an acceptable risk of bias. However, differences in study design, patient population, and treatment regimens should be considered when interpreting the results. Although research on treatment resistance and clinical outcomes in patients with cancer is increasing, much of the existing evidence remains based on resistant cell line models or animal studies rather than on patient cohorts. In addition, most studies primarily investigate miR-155 as a diagnostic biomarker (Hou et al., 2016; Shao et al., 2019; Wu et al., 2023; Wang F. et al., 2024), whereas fewer studies have explored its role in therapeutic targeting or treatment modulation (Liu et al., 2018; Tang et al., 2019). Since miR-155 may exhibit heterogeneous expression across cancers and treatment settings, further clinical research is required to understand its therapeutic significance (Hussain et al., 2025).

A further limitation of the included studies was the lack of consistent and detailed follow-up. Longitudinal post-treatment evaluation is crucial to determine whether changes in miR-155 expression are associated with significant clinical outcomes, including complete or partial response, PFS, DFS, OS, recurrence, and treatment resistance. However, several included studies had insufficient follow-up and lacked survival analyses, multivariate modeling, or sequential post-treatment miR-155 evaluations, limiting long-term assessment of the therapeutic relevance of miR-155.

## 7 Conclusion

The existing evidence suggests that miR-155 may be a context-dependent molecular marker, with its clinical significance varying by cancer type, biological specimen, timing of sample collection, and treatment approach. The substantial heterogeneity in miR-155 expression and its associations with treatment-related outcomes further indicates that its role in therapeutic response remains incompletely defined. Future research should prioritize prospective clinical validation using standardized methods for specimen collection, miR-155 measurement, normalization, and reporting, together with harmonized definitions of treatment response, resistance, and disease monitoring outcomes. Such studies are needed to determine whether miR-155 has clinically meaningful and independently validated relevance with treatment-related outcomes within specific cancer subtypes and therapeutic settings.

## Data Availability

The original contributions presented in the study are included in the article, further inquiries can be directed to the corresponding authors.

## References

[B1] Abdul-Rahman S. Y. Shareef A. Jyothi S. R. Nayak P. P. Janney J. B. Singh G. (2026). miR-155 and the orchestration of cell-death evasion and chemoresistance in cancer: isoform-specific mechanisms and therapeutic opportunities. Cancer Treat. Res. Commun. 47, 101198. 10.1016/j.ctarc.2026.101198 41946306

[B2] Aghiorghiesei A. I. Mehterov N. Nutu A. Vladimirov B. Nonchev B. Kontos C. K. (2025). The oncogenic role of miR-155 on oral cancer progression and treatment strategies. Discov. Onc 16, 1941. 10.1007/s12672-025-03467-2 PMC12540949 41117897

[B3] Al-Hawary S. I. S. Abdalkareem Jasim S. Altalbawy F. M. A. Kumar A. Kaur H. Pramanik A. (2024). miRNAs in radiotherapy resistance of cancer; a comprehensive review. Cell Biochem. Biophys. 82, 1665–1679. 10.1007/s12013-024-01329-2 38805114

[B4] Anwar S. L. Tanjung D. S. Fitria M. S. Kartika A. I. Sari D. N. I. Rakhmina D. (2020). Dynamic changes of circulating Mir-155 expression and the potential application as a non-invasive biomarker in breast cancer. Asian Pac J. Cancer Prev. 21, 491–497. 10.31557/APJCP.2020.21.2.491 32102529 PMC7332147

[B5] Attathikhun M. Jurj A. Calin G. A. (2025). MicroRNAs as key regulators of cancer drug resistance: insights and future directions in chemotherapy, targeted-therapy, radiotherapy, and immunotherapy. Cancer Drug Resist 8, 64. 10.20517/cdr.2025.146 PMC12713195 41425255

[B6] Bayraktar R. Van Roosbroeck K. (2018). miR-155 in cancer drug resistance and as target for miRNA-based therapeutics. Cancer Metastasis Rev. 37, 33–44. 10.1007/s10555-017-9724-7 29282605

[B7] Bota M. Vlaia L. Jîjie A.-R. Marcovici I. Crişan F. Oancea C. (2024). Exploring synergistic interactions between natural compounds and conventional chemotherapeutic drugs in preclinical models of lung cancer. Pharmaceuticals 17, 598. 10.3390/ph17050598 PMC11123751 38794168

[B8] Boyd L. N. C. Ali M. Puik J. R. Comandatore A. Ginocchi L. Meijer L. L. (2024). Plasma miR-379 can predict treatment response to FOLFIRINOX and gemcitabine-nab-paclitaxel in advanced pancreatic cancer. J. Liq. Biopsy 5, 100152. 10.1016/j.jlb.2024.100152 PMC11863948 40027944

[B9] Bray F. Laversanne M. Sung H. Ferlay J. Siegel R. L. Soerjomataram I. (2024). Global cancer statistics 2022: GLOBOCAN estimates of incidence and mortality worldwide for 36 cancers in 185 countries. CA A Cancer J. Clin. 74, 229–263. 10.3322/caac.21834 38572751

[B10] Campennì A. Aguennouz M. Siracusa M. Alibrandi A. Polito F. Oteri R. (2022). Thyroid cancer persistence in patients with unreliable Thyroglobulin measurement: circulating microRNA as candidate alternative biomarkers. Cancers 14, 5620. 10.3390/cancers14225620 PMC9688692 36428713

[B11] Chen J. Zhang H. Li X. Hu M. (2024). Correlations of high miRNA expressions with traditional proteins and prognosis of breast cancer. Rev. Romana Med. Lab. 32, 229–236. 10.2478/rrlm-2024-0018

[B12] Coletta R. D. Yeudall W. A. Salo T. (2024). Current trends on prevalence, risk factors and prevention of oral cancer. Front. Oral. Health 5, 1505833. 10.3389/froh.2024.1505833 PMC11599248 39606098

[B13] Darzi M. M. Neamati N. Sadeghi F. Bijani A. Moudi E. (2024). An intricate relationship between miR-155-5p expression and oxidative stress in bladder cancer patients treated with calmette-guerin immunotherapy. Int. J. Mol. Cell Med. 13 (2), 186–197. 10.22088/IJMCM.BUMS.13.2.186 PMC11344563 39184817

[B14] Dhuri K. Pradeep S. P. Shi J. Anastasiadou E. Slack F. J. Gupta A. (2022). Simultaneous targeting of multiple oncomiRs with phosphorothioate or PNA-based Anti-miRs in lymphoma cell lines. Pharm. Res. 39, 2709–2720. 10.1007/s11095-022-03383-y PMC9879158 36071352

[B15] Di Martino E. Smith L. Bradley S. H. Hemphill S. Wright J. Renzi C. (2022). Incidence trends for twelve cancers in younger adults—a rapid review. Br. J. Cancer 126, 1374–1386. 10.1038/s41416-022-01704-x PMC9090760 35132237

[B16] Dogra P. Shinglot V. Ruiz-Ramírez J. Cave J. Butner J. D. Schiavone C. (2024). Translational modeling-based evidence for enhanced efficacy of standard-of-care drugs in combination with anti-microRNA-155 in non-small-cell lung cancer. Mol. Cancer 23, 156. 10.1186/s12943-024-02060-5 PMC11295620 39095771

[B17] Due H. Schönherz A. A. Ryø L. Primo M. N. Jespersen D. S. Thomsen E. A. (2019). MicroRNA-155 controls vincristine sensitivity and predicts superior clinical outcome in diffuse large B-cell lymphoma. Blood Adv. 3, 1185–1196. 10.1182/bloodadvances.2018029660 30967394 PMC6457225

[B18] Due H. Brøndum R. F. Young K. H. Bøgsted M. Dybkær K. (2020). MicroRNAs associated to single drug components of R-CHOP identifies diffuse large B-cell lymphoma patients with poor outcome and adds prognostic value to the international prognostic index. BMC Cancer 20, 237. 10.1186/s12885-020-6643-8 32192453 PMC7082970

[B19] El‐Khazragy N. Noshi M. A. Abdel‐Malak C. Zahran R. F. Swellam M. (2019). miRNA‐155 and miRNA‐181a as prognostic biomarkers for pediatric acute lymphoblastic leukemia. J Cell. Biochem. 120, 6315–6321. 10.1002/jcb.27918 30362157

[B20] Esplugas R. Arenas M. Serra N. Bellés M. Bonet M. Gascón M. (2019). Effect of radiotherapy on the expression of cardiovascular disease-related miRNA-146a, -155, -221 and -222 in blood of women with breast cancer. PLoS ONE 14, e0217443. 10.1371/journal.pone.0217443 31150454 PMC6544229

[B21] Fawzy M. Ibrahiem A. Bayomy N. Makhdoom A. Alanazi K. Alanazi A. (2023). MicroRNA-155 and disease-related immunohistochemical parameters in cutaneous melanoma. Diagnostics 13, 1205. 10.3390/diagnostics13061205 PMC10047208 36980512

[B22] Grillone K. Caridà G. Luciano F. Cordua A. Di Martino M. T. Tagliaferri P. (2024). A systematic review of non-coding RNA therapeutics in early clinical trials: a new perspective against cancer. J. Transl. Med. 22, 731. 10.1186/s12967-024-05554-4 PMC11301835 39103911

[B23] Hanafi A. R. Jayusman A. M. Alfasunu S. Sadewa A. H. Pramono D. Heriyanto D. S. (2020). Serum MiRNA as predictive and prognosis biomarker in advanced stage non-small cell lung cancer in Indonesia. Zhongguo Fei Ai Za Zhi 23, 321–332. 10.3779/j.issn.1009-3419.2020.104.02 32283582 PMC7260391

[B24] Hou Y. Wang J. Wang X. Shi S. Wang W. Chen Z. (2016). Appraising MicroRNA-155 as a noninvasive diagnostic biomarker for cancer detection: a meta-analysis. Medicine 95, e2450. 10.1097/MD.0000000000002450 26765436 PMC4718262

[B25] Huang W. Kuo S. Kuo Y. Lin C. (2022). miR‐155‐regulated mTOR and toll‐like receptor 5 in gastric diffuse large b‐cell lymphoma. Cancer Med. 11, 555–570. 10.1002/cam4.4466 PMC8817081 34913612

[B26] Hussain Q. M. Al-Hussainy A. F. Sanghvi G. Roopashree R. Kashyap A. Anand D. A. (2025). Dual role of miR-155 and exosomal miR-155 in tumor angiogenesis: implications for cancer progression and therapy. Eur. J. Med. Res. 30, 393. 10.1186/s40001-025-02618-z PMC12087080 40383762

[B27] Kalkusova K. Taborska P. Stakheev D. Smrz D. (2022). The role of miR-155 in antitumor immunity. Cancers 14, 5414. 10.3390/cancers14215414 PMC9659277 36358832

[B28] Kousar K. Ahmad T. Abduh M. S. Kanwal B. Shah S. S. Naseer F. (2022). miRNAs in regulation of tumor microenvironment, chemotherapy resistance, immunotherapy modulation and miRNA therapeutics in cancer. IJMS 23, 13822. 10.3390/ijms232213822 PMC9699074 36430305

[B29] Lei Z. Tian Q. Teng Q. Wurpel J. N. D. Zeng L. Pan Y. (2023). Understanding and targeting resistance mechanisms in cancer. MedComm 4, e265. 10.1002/mco2.265 PMC10203373 37229486

[B30] Liu K. Zhao K. Wang L. Sun E. (2018). Prognostic value of microRNA-155 in human carcinomas: an updated meta-analysis. Clin. Chim. Acta 479, 171–180. 10.1016/j.cca.2018.01.036 29366836

[B31] Liu B. Su F. Lv X. Zhang W. Shang X. Zhang Y. (2019). Serum microRNA-21 predicted treatment outcome and survival in HER2-positive breast cancer patients receiving neoadjuvant chemotherapy combined with trastuzumab. Cancer Chemother. Pharmacol. 84, 1039–1049. 10.1007/s00280-019-03937-9 31482230

[B32] Papadaki C. Monastirioti A. Rounis K. Makrakis D. Kalbakis K. Nikolaou C. (2020). Circulating MicroRNAs regulating DNA damage response and responsiveness to cisplatin in the prognosis of patients with non-small cell lung cancer treated with first-line platinum chemotherapy. Cancers 12, 1282. 10.3390/cancers12051282 32438598 PMC7281609

[B33] Pathak S. Grillo A. R. Scarpa M. Brun P. D’Incà R. Nai L. (2015a). MiR-155 modulates the inflammatory phenotype of intestinal myofibroblasts by targeting SOCS1 in ulcerative colitis. Exp. Mol. Med. 47, e164. 10.1038/emm.2015.21 25998827 PMC4454995

[B34] Pathak S. Meng W.-J. Nandy S. K. Ping J. Bisgin A. Helmfors L. (2015b). Radiation and SN38 treatments modulate the expression of microRNAs, cytokines and chemokines in colon cancer cells in a p53-directed manner. Oncotarget 6, 44758–44780. 10.18632/oncotarget.5815 26556872 PMC4792590

[B35] Pavlíková L. Šereš M. Breier A. Sulová Z. (2022). The roles of microRNAs in cancer multidrug resistance. Cancers 14, 1090. 10.3390/cancers14041090 PMC8870231 35205839

[B36] Qian H. Maghsoudloo M. Kaboli P. J. Babaeizad A. Cui Y. Fu J. (2024). Decoding the promise and challenges of miRNA-Based cancer therapies: an essential update on miR-21, miR-34, and miR-155. Int. J. Med. Sci. 21, 2781–2798. 10.7150/ijms.102123 PMC11539376 39512697

[B37] Raue R. Frank A.-C. Syed S. N. Brüne B. (2021). Therapeutic targeting of MicroRNAs in the tumor microenvironment. IJMS 22, 2210. 10.3390/ijms22042210 PMC7926641 33672261

[B38] Sazonova E. V. Kopeina G. S. Imyanitov E. N. Zhivotovsky B. (2021). Platinum drugs and taxanes: can we overcome resistance? Cell Death Discov. 7, 155. 10.1038/s41420-021-00554-5 PMC8257727 34226520

[B39] Sedeta E. Sung H. Laversanne M. Bray F. Jemal A. (2023). Recent mortality patterns and time trends for the major cancers in 47 countries worldwide. Cancer Epidemiol. Biomarkers and Prev. 32, 894–905. 10.1158/1055-9965.EPI-22-1133 37195435

[B40] Shao C. Yang F. Qin Z. Jing X. Shu Y. Shen H. (2019). The value of miR-155 as a biomarker for the diagnosis and prognosis of lung cancer: a systematic review with meta-analysis. BMC Cancer 19, 1103. 10.1186/s12885-019-6297-6 31727002 PMC6854776

[B41] Shen M. Chen T. Li X. Zhao S. Zhang X. Zheng L. (2024). The role of miR-155 in urologic malignancies. Biomed. and Pharmacother. 174, 116412. 10.1016/j.biopha.2024.116412 38520867

[B42] Tang L. Peng Y. Li C. Jiang H. Mei H. Hu Y. (2019). Prognostic and clinicopathological significance of MiR-155 in hematologic malignancies: a systematic review and meta-analysis. J. Cancer 10, 654–664. 10.7150/jca.28537 30719163 PMC6360418

[B43] Thomopoulou K. Papadaki C. Monastirioti A. Koronakis G. Mala A. Kalapanida D. (2021). MicroRNAs regulating tumor immune response in the prediction of the outcome in patients with breast cancer. Front. Mol. Biosci. 8, 668534. 10.3389/fmolb.2021.668534 PMC8220200 34179081

[B44] Tili E. Michaille J.-J. Croce C. M. (2026). miR-155 aberrant expression impairs tumor rejection because of its targeting of ICOSL and multiple pathways implicated in the antitumor response. Pharmacol. Rev. 78, 100088. 10.1016/j.pharmr.2025.100088 41289737 PMC12881686

[B45] Tsotridou E. Georgiou E. Tragiannidis A. Avgeros C. Tzimagiorgis G. Lambrou M. (2023). miRNAs as predictive biomarkers of response to treatment in pediatric patients with acute lymphoblastic leukemia. Oncol. Lett. 27, 71. 10.3892/ol.2023.14204 38192661 PMC10773203

[B46] Wang J. Wang Q. Guan Y. Sun Y. Wang X. Lively K. (2022). Breast cancer cell–derived microRNA-155 suppresses tumor progression *via* enhancing immune cell recruitment and antitumor function. J. Clin. Investigation 132, e157248. 10.1172/JCI157248 PMC9525116 35925680

[B47] Wang F. Wang J. Zhang H. Fu B. Zhang Y. Jia Q. (2024a). Diagnostic value of circulating miR-155 for breast cancer: a meta-analysis. Front. Oncol. 14, 1374674. 10.3389/fonc.2024.1374674 PMC10999615 38590648

[B48] Wang Z. Wang H. Zhou S. Mao J. Zhan Z. Duan S. (2024b). miRNA interplay: mechanisms and therapeutic interventions in cancer. MedComm – Oncol. 3, e93. 10.1002/mog2.93

[B49] Wang M. Gao X. Zhang L. (2025). Recent global patterns in skin cancer incidence, mortality, and prevalence. Chin. Med. J. 138, 185–192. 10.1097/CM9.0000000000003416 PMC11745855 39682020

[B50] Wu Y. Hong Q. Lu F. Zhang Z. Li J. Nie Z. (2023). The diagnostic and prognostic value of miR-155 in cancers: an updated meta-analysis. Mol. Diagn Ther. 27, 283–301. 10.1007/s40291-023-00641-6 36939982

[B51] Yusoh N. A. Ahmad H. Vallis K. A. Gill M. R. (2025). Advances in platinum-based cancer therapy: overcoming platinum resistance through rational combinatorial strategies. Med. Oncol. 42, 262. 10.1007/s12032-025-02812-3 40518502 PMC12167724

[B52] Zafar A. Khatoon S. Khan M. J. Abu J. Naeem A. (2025). Advancements and limitations in traditional anti-cancer therapies: a comprehensive review of surgery, chemotherapy, radiation therapy, and hormonal therapy. Discov. Onc 16, 607. 10.1007/s12672-025-02198-8 PMC12021777 40272602

[B53] Zang Y.-S. Zhong Y.-F. Fang Z. Li B. An J. (2012). MiR-155 inhibits the sensitivity of lung cancer cells to cisplatin *via* negative regulation of Apaf-1 expression. Cancer Gene Ther. 19, 773–778. 10.1038/cgt.2012.60 22996741

[B54] Zare N. Haghjooy Javanmard S. Mehrzad V. Eskandari N. Kefayat A. (2019). Evaluation of exosomal miR-155, let-7g and let-7i levels as a potential noninvasive biomarker among refractory/relapsed patients, responsive patients and patients receiving R-CHOP. Leukemia and Lymphoma 60, 1877–1889. 10.1080/10428194.2018.1563692 30714442

[B55] Zhang Z. Zhang L. Yu G. Sun Z. Wang T. Tian X. (2020). Exosomal miR-1246 and miR-155 as predictive and prognostic biomarkers for trastuzumab-based therapy resistance in HER2-positive breast cancer. Cancer Chemother. Pharmacol. 86, 761–772. 10.1007/s00280-020-04168-z 33068176

[B56] Zheng Z. Sun R. Zhao H.-J. Fu D. Zhong H.-J. Weng X.-Q. (2019). MiR155 sensitized B-lymphoma cells to anti-PD-L1 antibody *via* PD-1/PD-L1-mediated lymphoma cell interaction with CD8+T cells. Mol. Cancer 18, 54. 10.1186/s12943-019-0977-3 30925928 PMC6441197

